# Evaluation of GABA-Producing Fermented Whey Formulations: From Strain Selection to Raspberry-Enriched Beverages with Psychobiotic Potential

**DOI:** 10.3390/foods14162762

**Published:** 2025-08-08

**Authors:** Mariano Del Toro-Barbosa, Tlalli Uribe-Velázquez, Alejandra Hurtado-Romero, María Fernanda Rosales-De la Cruz, Danay Carrillo-Nieves, Luis Eduardo Garcia-Amezquita, Tomás García-Cayuela

**Affiliations:** 1 Tecnologico de Monterrey, Escuela de Ingeniería y Ciencias, Avenida General Ramón Corona 2514, Zapopan 45138, Mexico; 2 Tecnologico de Monterrey, Escuela de Ingeniería y Ciencias, Avenida Eugenio Garza Sada 2501, Monterrey 64849, Mexico

**Keywords:** gamma-aminobutyric acid, probiotic viability, phenolic stability, whey valorization, gut–brain axis, functional beverage, fermented food

## Abstract

Certain probiotic strains have been proposed to alleviate mental health conditions, such as anxiety and stress, by modulating the gut–microbiota–brain axis through the production of metabolites like gamma-aminobutyric acid (GABA). This study evaluated kefir-derived microbial strains for their GABA-producing capacity in mono- and co-culture systems using whey as the growth substrate. Based on the screening results, two microbial consortia were selected to develop fermented whey beverages with raspberry (FWF-R1 and FWF-R2). These beverages were characterized for their technological and functional properties over 21 days of refrigerated storage and following gastrointestinal digestion. Both formulations maintained stable acidity and showed a slight increase in viscosity during storage. The microbial counts remained above 8.5 log colony-forming units/mL, with high post-digestion viability, confirming their probiotic potential. The GABA levels increased progressively during storage, reaching 2.67 mM in FWF-R1 and 4.65 mM in FWF-R2, with recovery rates of 40–45% after digestion. The total phenolic content decreased moderately during storage but increased ~5-fold after digestion; the total anthocyanins declined by up to 70%. FWF-R2 achieved higher sensory acceptability and was preferred by 58% of consumers, emerging as the most promising formulation. These findings highlight the psychobiotic potential of these beverages and support the sustainable valorization of dairy and fruit by-products.

## 1. Introduction

Mental health disorders are among the leading causes of disability worldwide, with anxiety and depression being the most prevalent conditions [1]. Anxiety disorders are characterized by persistent and excessive fear, worry, or nervousness that is disproportionate to the actual threats and interferes with cognitive and executive functions [2,3]. Although the etiology of anxiety is not fully understood, several neurobiological mechanisms have been implicated, including altered neurotransmitter signaling, particularly involving gamma-aminobutyric acid (GABA), the principal inhibitory neurotransmitter in the central nervous system (CNS), as well as dysfunctions in the gut–microbiota–brain axis (GMBA) [4,5].

The GMBA is a complex, bidirectional communication system linking the CNS and the gastrointestinal tract through vagal, endocrine, and immune pathways [6]. Beyond its role in digestion, the GMBA also influences emotional and cognitive processes. The gut microbiota plays a key role in regulating this axis by producing bioactive compounds that reduce inflammation, modulate cortisol levels, and influence mood and behavior [7]. Among these microbial metabolites, neurotransmitters, such as GABA and short-chain fatty acids (SCFAs), have received increasing attention for their ability to modulate brain function. Consequently, novel therapeutic strategies targeting the GMBA may serve as complementary tools to conventional anxiety treatments.

Although pharmacological therapies can be effective, their side effects and low adherence underscore the need for alternative or complementary approaches. In this context, psychobiotics, defined as live microorganisms or their metabolites that confer mental health benefits when consumed in adequate amounts, have emerged as promising candidates [8]. Certain microorganisms, including lactic acid bacteria (LAB), can produce GABA via glutamate decarboxylation [9]. Numerous studies have explored GABA enrichment through fermentation in various food matrices, including dairy, cereal-based, fruit-based, and plant-based systems [10]. However, most of these investigations have focused solely on GABA quantification prior to consumption, with limited attention paid to its gastrointestinal stability in complex food systems.

In previous work, our research group isolated and characterized microbial strains from Mexican kefir grains with confirmed probiotic potential and GABA-producing capacity [11]. These kefir-derived strains represent promising psychobiotic candidates for the development of functional fermented foods targeting mental well-being. Moreover, the development of such foods can be strategically aligned with sustainability goals, particularly through the valorization of agro-industrial by-products [12].

Fermentation is a well-established biotechnological tool used to enhance the nutritional and functional value of foods, while also promoting sustainability through the valorization of agro-industrial waste streams [13]. In this context, Jalisco, one of Mexico’s major agro-industrial regions, produces large volumes of cheese and berries, particularly raspberries [14,15]. Two of the region’s most pressing environmental issues are the underutilization of whey, a dairy by-product rich in proteins and lactose [16], and postharvest losses of raspberries, which are rich in anthocyanins, polyphenols, and dietary fiber [17]. The combination of these two matrices presents a highly promising substrate for the development of fermented beverages with both health-promoting and sustainability benefits.

Using kefir-derived microbial strains to ferment a whey–raspberry matrix could thus help address both environmental and mental health challenges. While whey-based fermented beverages have been explored for their nutritional, technological, and bioactive properties [18,19,20], formulations combining whey and raspberry remain poorly documented. Scientific evidence on the synergistic effects of whey and raspberry ingredients, their influence on GABA production, and their functional stability during digestion is still limited.

Therefore, this study aimed to (i) valorize agro-industrial by-products, specifically whey through fermentation and raspberry residues as functional ingredients, and (ii) develop fermented beverages with potential mental health benefits using GABA-producing strains isolated from kefir. To achieve this, we characterized the beverages’ physicochemical properties, nutritional composition, GABA content, microbial viability, and bioactive compound profiles. Their stability during in vitro digestion and their sensory acceptability were also evaluated.

## 2. Materials and Methods

### 2.1. Whey: Processing and Physicochemical and Proximate Characterization

Whey was kindly provided by Grupo Serrano (Lagos de Moreno, Mexico) and was obtained from the production of various cheeses using pasteurized skim milk. Upon arrival at the laboratory, the whey was transported in a cooler, aliquoted into containers, and stored at −20 °C until use. Before fermentation, it was thawed and pasteurized in a water bath at 72 °C for 15 s in sealed glass containers. After pasteurization, the jars were cooled and stored at 4 °C under aseptic conditions, and the whey was used within 24 h. These pasteurization and storage conditions were previously validated to ensure the absence of spoilage microorganisms.

Physicochemical analyses were performed at room temperature on the pasteurized whey. The pH was measured using a calibrated pH meter (Horiba LAQUAact-PH110-K, Kyoto, Japan), while the titratable acidity was determined according to the Mexican Official Standard NOM-155-SCFI-2012 [21] using 0.1 N NaOH. The viscosity was assessed with a CVP-8S viscometer (CScientific, Beijing, China), maintaining the torque values within the 20–80% range as recommended by the manufacturer. The water-holding capacity (WHC) was evaluated by centrifuging 20 g of whey at 1500× *g* for 15 min and recording the weight of the supernatant [22].

The proximate composition was determined using official AOAC methods. The protein content was quantified via the Kjeldahl method (AOAC 920.152), and the lipid content was measured using a modified Goldfisch extraction with petroleum ether (AOAC 960.39). The total dietary fiber was assessed through an enzymatic–gravimetric analysis (AOAC 2011.25), and the ash content was measured by incineration (AOAC 940.26). The digestible carbohydrates were calculated by the difference.

### 2.2. Bacterial Strains and Fermentation Conditions in Whey

To evaluate fermentation in whey, mono-cultures of ten strains from our BIOTEC collection were used. These strains were previously isolated from traditional Mexican kefir and characterized for their probiotic potential [11]. In addition, three commercial probiotic strains were included: *Lactobacillus acidophilus* LA3, *Lacticaseibacillus rhamnosus* GG, and *Lactiplantibacillus plantarum* 299v.

All strains were stored at −80 °C and subcultured twice under aerobic conditions before use. *Lactococcus lactis* strains were grown in M17 broth (BD Difco, Sparks, MD, USA) supplemented with 0.5% (*w*/*v*) lactose (LM17); *Kluyveromyces lactis* strains were cultured in yeast extract–dextrose–peptone (YPD) medium; and remaining strains were propagated in Man–Rogosa–Sharpe (MRS) broth (BD Difco). Each subculture was incubated at 30 °C for 24 h. After second propagation, cultures were centrifuged (3000× *g*, 10 min, 4 °C), washed, and resuspended in sterile 0.85% (*w*/*v*) saline solution.

The resuspended cultures were inoculated at 2% (*v*/*v*) into pasteurized whey and incubated aerobically at 30 °C for 24 h. Microbial viability, expressed as colony-forming units (CFU)/mL, was assessed at beginning and end of fermentation by serial dilutions and plating on respective media, solidified with 1.5% bacteriological agar (BD Difco). Final pH was also recorded after fermentation.

### 2.3. Determination of GABA in Whey Cultures

The extracellular GABA levels in the whey cultures were quantified using a GABase enzymatic assay (Sigma-Aldrich, St. Louis, MO, USA), following a previously described protocol [23] with minor modifications. GABase is a coupled enzyme system consisting of GABA aminotransferase and succinic semialdehyde dehydrogenase, which convert GABA into succinic acid with concomitant NADPH production, measurable by spectrophotometry.

Following fermentation, the cultures were centrifuged (3000× *g*, 10 min, 4 °C), and the supernatants were filtered through 0.45 µm syringe filters (Merck Millipore, Billerica, MA, USA). For the assay, the filtrate (10 µL) was combined with 90 µL of reaction buffer in a 96-well microplate and incubated at 30 °C for 120 min. The buffer contained 80 mM Tris–HCl (pH 9.0), 750 mM sodium sulfate, 10 mM dithiothreitol, 1.4 mM NADP^+^, 2 mM α-ketoglutarate, and 100 µg GABase per well. The absorbance was recorded at 340 nm using a microplate reader (Varioskan Lux, Thermo Fisher Scientific, Waltham, MA, USA). A blank was prepared by omitting the GABase enzyme. The GABA concentrations (mM) were determined using a standard calibration curve.

### 2.4. Strain Selection and GABA-Based Evaluation for Beverage Formulation

Each mono-culture fermented in whey was evaluated through an internal sensory screening by a panel of food product specialists (*n* = 9) within the research group. The attributes evaluated included the acidity, sweetness, texture, and overall acceptability (Appendix A). Based on these results and an iterative testing of the strain combinations, three microbial consortia were selected for further study: Mix 1, Mix 2, and Mix 3. All the formulations were inoculated at a total concentration of 4% (*v*/*v*); however, the relative proportions of each microorganism varied across the consortia, as shown in Table 1. After 24 h of fermentation at 30 °C, the GABA concentrations in the whey formulations were measured as described in Section 2.3.

### 2.5. Raspberry Powder: Processing and Physicochemical, Proximate, and Bioactive Characterization

Freeze-dried raspberry powder was included in the final beverage formulation (Section 2.6) as a functional ingredient due to its high bioactive content. Non-commercial raspberries (*Rubus idaeus* L., cv. Adelita) were provided by BlueDrop Berries (Ahualulco de Mercado, Mexico). Upon receipt, the fruits were washed, disinfected, and stored at −80 °C until processing. Prior to freeze-drying, their basic physicochemical properties were evaluated as described previously [24], including the size calibration, moisture content, total soluble solids (°Brix), pH, and titratable acidity.

The raspberries were freeze-dried (−83 °C, 0.035 mbar) using a FreeZone 4.5 unit (Labconco, Kansas City, MO, USA), ground with an analytical mill (IKA A10 basic, IKA-Werke, Staufen im Breisgau, Germany), sieved to obtain a particle size of 105 µm, and stored at −20 °C in sealed containers. Their proximate composition was determined using the same methods described in Section 2.1. The total phenolic content (TPC) and total anthocyanin content (TAC) were assessed following the protocols in Section 2.7.5.

### 2.6. Final Beverage Formulation and Proximate Composition

A base formulation of 75% fermented whey and 25% ultra-pasteurized skim milk was selected to enhance the sensory appeal and mimic the texture of drinkable yogurt, while retaining the nutritional and functional benefits of the whey matrix.

Based on their GABA production, mixed cultures from Mix 1 (BIOTEC007 + LA3) and Mix 3 (BIOTEC007, BIOTEC009, BIOTEC012, BIOTEC014, and LA3) were chosen to ferment the whey at 30 °C for 24 h. After fermentation, the final beverages were prepared by blending 75% fermented whey with 0.8% (*w*/*v*) commercial sweetener (Splenda, Tultitlán, Mexico; containing 1.2% sucralose), 2.5% (*w*/*v*) agave inulin (Hacienda de Oro de Agave, Amatitán, Mexico), and 2.5% (*w*/*v*) freeze-dried raspberry powder. The remaining 25% consisted of ultra-pasteurized skim milk (1% fat; Grupo Lala, Gómez Palacio, Mexico) preheated to dissolve 0.25% (*w*/*v*) carrageenan (Sosa, Barcelona, Spain). After cooling, the milk phase was gradually added to the whey blend under constant stirring to ensure homogeneity. All the ingredient concentrations were calculated relative to the total final beverage volume.

The resulting fermented whey formulations with raspberry (FWF-R) were designated FWF-R1 and FWF-R2, with FWF-R1 fermented by mixed culture 1 (Mix 1), and FWF-R2 by mixed culture 3 (Mix 3), according to Table 1. Their proximate compositions were analyzed as described in Section 2.1.

### 2.7. Shelf-Life Study and In Vitro Gastrointestinal Stability of Functional Fermented Beverages

Shelf-life evaluation of FWF-R1 and FWF-R2 was conducted at five time points: day 0 (formulation), and days 1, 7, 14, and 21 of refrigerated storage (4 °C). At each time point, physicochemical properties, microbial viability, GABA concentration, and total bioactive content were assessed. Additionally, samples underwent in vitro gastrointestinal digestion, followed by analysis of microbial viability and stability of GABA and bioactive compounds.

#### 2.7.1. Physicochemical Parameters

pH, titratable acidity, viscosity, and WHC were measured as described in Section 2.1. Soluble solids (°Brix at 25 °C) were measured using digital refractometer (Hanna Instruments, Smithfield, RI, USA).

#### 2.7.2. In Vitro Gastrointestinal Digestion

Digestion was performed following the INFOGEST protocol [25], with slight modifications. Simulated digestive fluids for the oral, gastric, and intestinal phases were prepared using NaCl (120 g/L), KCl (46.7 g/L), MgCl_2_⋅6H_2_O (30 g/L), KH_2_PO_4_ (68 g/L), and NaHCO_3_ (84 g/L). The fresh samples (5 mL) were initially mixed 1:1 (*v*/*v*) with the simulated salivary fluid containing α-amylase (75 U/mL), adjusted to pH 7, and incubated for 2 min. The resulting oral bolus was then combined 1:1 (*v*/*v*) with the simulated gastric fluid containing pepsin (2000 U/mL), adjusted to pH 3, and incubated for 2 h. Finally, the gastric chyme was mixed 1:1 (*v*/*v*) with the simulated intestinal fluid supplemented with bile salts (10 mM) and pancreatin (100 U/mL), adjusted to pH 7.0, and incubated for an additional 2 h. All the incubations were carried out at 37 °C with constant agitation (100 rpm) using an Ecotron shaking incubator (Infors, Annapolis Junction, MD, USA). Post-digestion aliquots were collected for microbial viability and GABA quantification. The remaining digesta samples were freeze-dried and stored at −80 °C for further bioactive compound analysis.

#### 2.7.3. Microbial Viability

Pre- and post-digestion samples were serially diluted in sterile 0.85% (*w*/*v*) saline solution and plated on MRS agar. Plates were incubated at 30 °C for 48–72 h, and results expressed as log CFU/mL.

#### 2.7.4. GABA Concentration

GABA levels (mM) before and after digestion were quantified using GABase enzymatic assay described in Section 2.3.

#### 2.7.5. Total Bioactive Compounds

Samples were freeze-dried and extracted with methanol/water (50:50, *v*/*v*) as described previously [26]. TPC was determined using Folin–Ciocalteu assay [27] and TAC by pH-differential method [28]. TPC was reported as mg gallic acid equivalents (GAE)/100 mL and TAC as mg cyanidin-3-glucoside equivalents (C3G)/100 mL.

### 2.8. Sensory Evaluation of Functional Fermented Beverages

A sensory evaluation was conducted with 113 untrained panelists (56 female, 57 male), aged 14–46 years, and all regular dairy product consumers. The study was approved by the Institutional Research Ethics Committee (CIEI, protocol CA-EIC-2406-02), and informed consent was obtained from all the participants following a standardized briefing.

The panelists evaluated three samples: FWF-R1, FWF-R2, and one unfermented whey formulation containing raspberry as the control (WF-RC). The WF-RC was prepared using the same formulation and processing conditions as FWF-R1 and FWF-R2 (Section 2.6), but without microbial inoculation or fermentation. Each 20 mL sample was served at 10 °C in coded plastic cups under standardized conditions. The sensory attributes included the appearance, color, aroma, texture, sweetness, acidity, dairy flavor, and fruitiness, using a 9-point hedonic scale (1 = “dislike very much”; 9 = “like very much”). The acceptability index for each attribute was calculated as a percentage of the maximum score [24]. The participants also identified their preferred sample and answered questions regarding the perceived importance of mental health, their current consumption of products with mental health benefits, and willingness to purchase such products.

### 2.9. Statistical Analysis

All experiments were performed in triplicate, and results were presented as mean ± standard deviation. Statistical analyses were conducted using Minitab Software (version 21.4; Minitab LLC, State College, PA, USA). One-way analysis of variance (ANOVA) was used to evaluate differences among multiple groups, followed by Tukey’s test for post hoc comparisons. For comparisons between two groups, paired Student’s *t*-tests were applied. Statistical significance was established at *p* ≤ 0.05.

## 3. Results

### 3.1. Physicochemical and Proximate Characterization of Whey

The pasteurized skim whey used as the fermentation substrate exhibited a slightly acidic pH (5.70 ± 0.02) and a titratable acidity of 1.68 ± 0.07 g/L (Table 2). Its low viscosity and limited WHC were consistent with the low solids content typical of diluted dairy liquids.

In terms of its proximate composition, the whey was high in carbohydrates (4.59 g/100 g), and low in protein and fat. The ash content was 0.51 g/100 g, and no dietary fiber was detected. These features support its suitability as a fermentation medium for microbial growth.

### 3.2. Fermentation of Whey by Mono-Cultures

#### 3.2.1. Microbial Counts and Acidification

The growth of each strain in the pasteurized whey was assessed by monitoring the CFU/mL and pH after 24 h of fermentation at 30 °C (Table 3). Most of the strains demonstrated viable growth, with significant increases in the microbial counts (*p* ≤ 0.05) observed in 10 of the 13 tested cultures. The three commercial strains (LA3, GG, and 299v) reached final counts above 8.9 log CFU/mL.

The acidification capacity varied notably among the strains, with the final pH values ranging from 4.03 to 5.73. The commercial strains achieved the lowest pH values, indicating strong acidification potential. In contrast, *Leuconostoc pseudomesenteroides* BIOTEC011 and *Kluyveromyces lactis* BIOTEC010 showed no acidification, presenting pH values comparable to the non-inoculated control (5.70). These results highlight the diverse metabolic responses and adaptation levels of the tested strains in whey.

#### 3.2.2. GABA Production by Mono-Cultures

**Figure 1:**
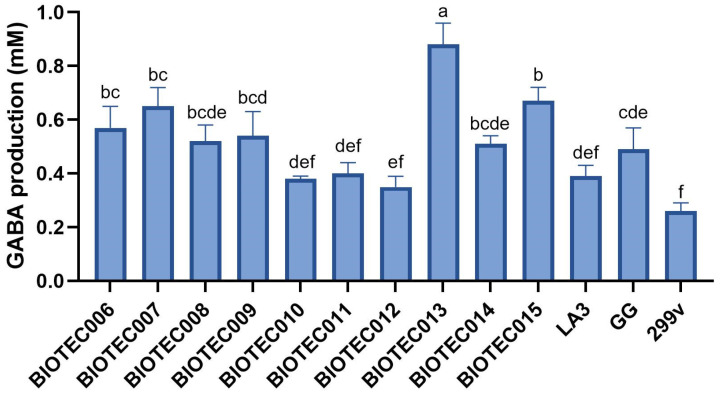
Gamma-aminobutyric acid (GABA) production by individual strains grown in pasteurized whey. Bars represent mean values, and error bars indicate standard deviations. Different letters indicate statistically significant differences between strains (*p* ≤ 0.05). Full microorganism names are provided in Table 3.

The GABA concentrations produced by the individual strains in the pasteurized whey are presented in Figure 1. All the strains produced detectable GABA levels, with statistically significant differences among them (*p* ≤ 0.05). The *Lactococcus lactis* strains yielded 0.52–0.65 mM GABA, with BIOTEC006 and BIOTEC007 showing moderate production, and BIOTEC008 slightly lower. *K. lactis* BIOTEC009 and BIOTEC010 produced 0.38–0.54 mM, similar to that of several bacterial strains. *L. pseudomesenteroides* BIOTEC011 and BIOTEC012 showed low production (≤0.40 mM), with no significant difference between them. Within *Lentilactobacillus*, BIOTEC013 produced the highest GABA concentration (0.88 ± 0.08 mM), while BIOTEC014 and BIOTEC015 showed moderate levels. Among the commercial strains, LA3 and GG had low production (0.39 and 0.49 mM), and 299v showed the lowest (0.26 ± 0.03 mM). These findings confirm strain-specific variability in GABA biosynthesis, regardless of the microbial taxonomy.

### 3.3. Strain Selection and Preliminary Formulation Assessment

#### 3.3.1. Sensory Screening of Mono and Mixed Cultures

Each mono-culture grown in pasteurized whey was first evaluated by a panel of food product specialists (*n* = 9) for general sensory attributes (Appendix A, Table A1). Among the *L. lactis* strains, BIOTEC007 was notable for its yogurt-like aroma and mildly acidic taste, yielding the familiar and pleasant profile of fermented dairy products. This strain was selected as the primary starter culture for the subsequent blends. Additional LAB strains were chosen for their unique sensory characteristics: BIOTEC012 offered a fruity aroma and cheese-like flavor reminiscent of fresh cheese, while BIOTEC014 presented a mild dairy aroma and a clean, neutral taste. Although *L. kefiri* BIOTEC013 exhibited the highest GABA production (Figure 1), it was excluded due to its strong astringency. The yeast *K. lactis* BIOTEC009 was included for its refreshing mouthfeel linked to slight gas production. The commercial strain *L. acidophilus* LA3 was selected for its balanced fruity and acidic profile.

To assess the sensory acceptability, three mixed cultures were evaluated for acidity, sweetness, texture, and overall perception using an acceptability index (Appendix A, Table A2). The best-performing combinations were as follows: Mix 1 (BIOTEC007 + LA3), Mix 2 (BIOTEC007 + BIOTEC012 + LA3), and Mix 3 (BIOTEC007 + BIOTEC009 + BIOTEC012 + BIOTEC014 + LA3). These were further analyzed for their GABA production.

#### 3.3.2. GABA Production by Mixed Cultures

**Figure 2:**
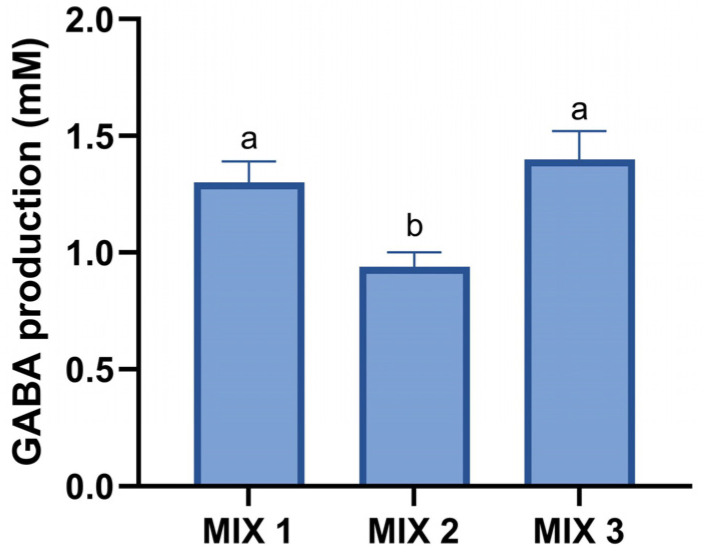
Gamma-aminobutyric acid (GABA) production by mixed cultures grown in pasteurized whey. Bars represent mean values, and error bars indicate standard deviations. Different letters indicate statistically significant differences between mixed cultures (*p* ≤ 0.05). The composition of each mixed culture is detailed in Table 1.

All the selected mixed cultures produced higher GABA levels than the individual strains. As shown in Figure 2, Mix 1 and Mix 3 reached the highest concentrations, 1.30 ± 0.09 mM and 1.40 ± 0.12 mM, respectively, while Mix 2 showed significantly lower production (0.94 ± 0.06 mM). Based on these results, Mix 1 and Mix 3 were chosen for the final development of fermented whey beverages.

### 3.4. Characterization of Raspberry Fruit and Powder

The physicochemical properties of the fresh raspberries are shown in Table 4. After freeze-drying, the resulting raspberry powder exhibited a high dietary fiber content (20.82 g/100 g dry weight [dw]), primarily insoluble (81%), with a smaller soluble fraction (19%). The powder also contained high levels of bioactive compounds, including TPC (9.21 mg/g dw) and TAC (2.64 mg/g dw), supporting its use as a functional ingredient in beverage formulations.

### 3.5. Proximate Composition and Physicochemical Properties of Functional Fermented Beverages

#### 3.5.1. Proximate Composition of Beverages

The proximate composition of the two fermented whey formulations with raspberry (FWF-R1 and FWF-R2) is presented in Table 5. No significant differences (*p* > 0.05) were found between the formulations. Both had low protein and fat levels, consistent with their whey and skim milk base. The dietary fiber content ranged from 0.97 to 1.12 g/100 g (fresh weight), primarily contributed by the freeze-dried raspberry powder and added inulin.

#### 3.5.2. Physicochemical Properties During Storage

The physicochemical changes in FWF-R1 and FWF-R2 during 21 days of refrigerated storage are shown in Table 6. The pH remained stable, with FWF-R2 consistently showing slightly higher values than FWF-R1. The titratable acidity fluctuated minimally, without a clear trend. The soluble solids increased modestly in both formulations, with earlier rises in FWF-R1 (day 1) and delayed increases in FWF-R2 (day 14); overall, FWF-R2 exhibited a higher °Brix. The viscosity increased progressively in both beverages, peaking at day 21. The WHC rose slightly on day 1 and remained stable thereafter, suggesting structural stabilization during storage.

### 3.6. Microbial Viability During Storage and After Digestion

**Figure 3:**
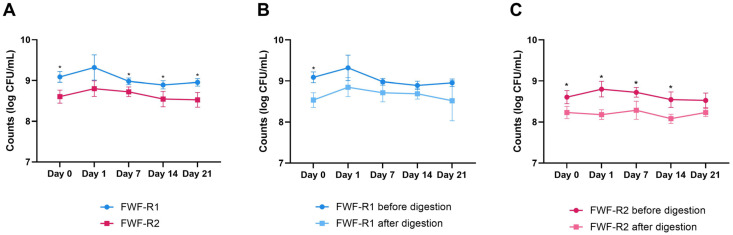
Viability of fermenting microorganisms in fermented whey formulations with raspberry (FWF-R1 and FWF-R2) during refrigerated storage and after in vitro gastrointestinal digestion. Microbial counts of FWF-R1 and FWF-R2 throughout 21 days of storage at 4 °C (**A**). Microbial counts of FWF-R1 before and after simulated gastrointestinal digestion at each storage time point (**B**). Microbial counts of FWF-R2 before and after digestion at each storage time point (**C**). Bars represent mean values, and error bars indicate standard deviations. No significant differences were observed in microbial viability during shelf-life for either formulation. Asterisks indicate significant differences (*p* ≤ 0.05) between samples at same storage time. FWF-R1 was fermented using mixed culture 1 (Mix 1), and FWF-R2 with mixed culture 3 (Mix 3). Microbial composition of each mixture is detailed in Table 1. CFU: colony forming units.

To facilitate viability monitoring across the strains, MRS agar was used, as the preliminary tests confirmed comparable growth for both the LAB and yeast strains. After fermentation (day 0), FWF-R1 reached 9.09 ± 0.13 log CFU/mL, and FWF-R2 8.61 ± 0.16 log CFU/mL (Figure 3A). This initial difference was maintained throughout the 21-day storage period, with the microbial counts remaining stable in both formulations.

The simulated gastrointestinal digestion at each time point showed no significant changes in the microbial viability (Figure 3B,C). For FWF-R1, the viability remained unchanged post-digestion. In FWF-R2, slight but significant decreases (0.3–0.5 log CFU/mL) were observed after digestion compared to the undigested samples. Despite this, both formulations retained viable counts between 8.23 and 8.52 log CFU/mL at the end of storage, confirming their probiotic potential and stability under digestive conditions.

### 3.7. GABA Content During Storage and After Digestion

**Figure 4:**
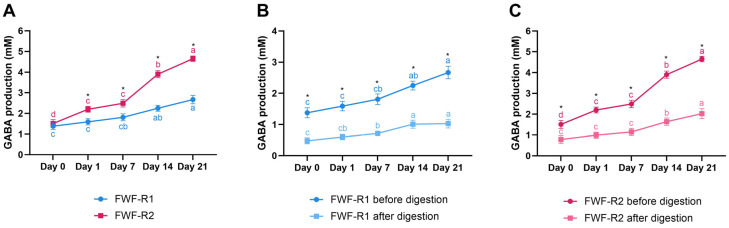
Gamma-aminobutyric acid (GABA) content in fermented whey formulations with raspberry (FWF-R1 and FWF-R2) during refrigerated storage and after in vitro gastrointestinal digestion. GABA content of FWF-R1 and FWF-R2 throughout 21 days of storage at 4 °C (**A**). GABA content of FWF-R1 before and after simulated gastrointestinal digestion at each storage time point (**B**). GABA content of FWF-R2 before and after digestion at each storage time point (**C**). Bars represent mean values, and error bars indicate standard deviations. Different lowercase letters indicate significant differences (*p* ≤ 0.05) in GABA content during shelf-life. Asterisks indicate significant differences (*p* ≤ 0.05) between samples at same storage time. FWF-R1 was fermented using mixed culture 1 (Mix 1), and FWF-R2 with mixed culture 3 (Mix 3). Microbial composition of each mixture is detailed in Table 1.

Both FWF-R1 and FWF-R2 showed a progressive increase in the GABA concentration during refrigerated storage (Figure 4A). Significant differences between the formulations emerged from day 1 (*p* ≤ 0.05), with FWF-R2 consistently displaying higher levels than FWF-R1. By day 21, the peak concentrations reached 4.65 mM for FWF-R2 and 2.67 mM for FWF-R1. The GABA accumulation in FWF-R2 also showed a more rapid and sustained increase over time.

The simulated gastrointestinal digestion led to significant GABA losses in both formulations (*p* ≤ 0.05), averaging reductions of ~55% in FWF-R1 and ~60% in FWF-R2 (Figure 4B,C). The post-digestion GABA levels ranged from 0.48 to 1.03 mM for FWF-R1 and from 0.78 to 2.03 mM for FWF-R2 throughout the storage period.

### 3.8. Stability of Bioactive Compounds During Storage and After Digestion

**Figure 5:**
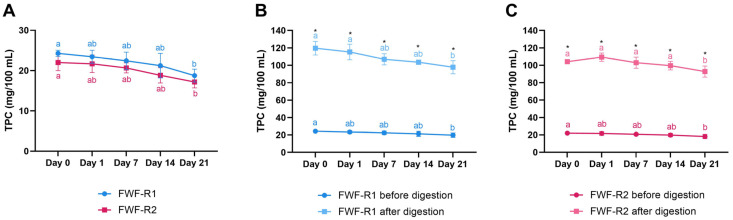
Total phenolic content (TPC) in fermented whey formulations with raspberry (FWF-R1 and FWF-R2) during refrigerated storage and after in vitro gastrointestinal digestion. TPC of FWF-R1 and FWF-R2 throughout 21 days of storage at 4 °C (**A**). TPC of FWF-R1 before and after simulated gastrointestinal digestion at each storage time point (**B**). TPC of FWF-R2 before and after digestion at each storage time point (**C**). Bars represent mean values, and error bars indicate standard deviations. Different lowercase letters indicate significant differences (*p* ≤ 0.05) in TPC during shelf-life. Asterisks indicate significant differences (*p* ≤ 0.05) between samples at same storage time. FWF-R1 was fermented using mixed culture 1 (Mix 1), and FWF-R2 with mixed culture 3 (Mix 3). Microbial composition of each mixture is detailed in Table 1. TPC expressed as mg of gallic acid equivalents per 100 mL.

Both FWF-R1 and FWF-R2 showed similar trends in their TPC during refrigerated storage (Figure 5A). The TPC declined significantly over 21 days (*p* ≤ 0.05), with reductions of ~23% in FWF-R1 (24.28 to 18.76 mg/100 mL) and ~22% in FWF-R2 (22.00 to 17.18 mg/100 mL). The simulated gastrointestinal digestion significantly increased the TPC compared to the undigested samples at all time points (*p* ≤ 0.05), with ~4.7-fold and ~5.1-fold increases in FWF-R1 and FWF-R2, respectively (Figure 5B,C). This suggests an enhanced release or transformation of phenolics during digestion, potentially improving their bioaccessibility.

**Figure 6:**
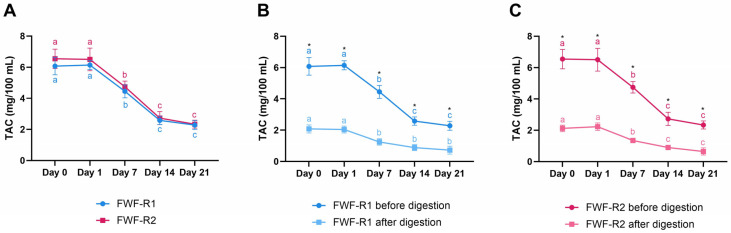
Total anthocyanin content (TAC) in fermented whey formulations with raspberry (FWF-R1 and FWF-R2) during refrigerated storage and after in vitro gastrointestinal digestion. TAC of FWF-R1 and FWF-R2 throughout 21 days of storage at 4 °C (**A**). TAC of FWF-R1 before and after simulated gastrointestinal digestion at each storage time point (**B**). TAC of FWF-R2 before and after digestion at each storage time point (**C**). Bars represent mean values, and error bars indicate standard deviations. Different lowercase letters indicate significant differences (*p* ≤ 0.05) in TAC during shelf-life. Asterisks indicate significant differences (*p* ≤ 0.05) between samples at same storage time. FWF-R1 was fermented using mixed culture 1 (Mix 1), and FWF-R2 with mixed culture 3 (Mix 3). Microbial composition of each mixture is detailed in Table 1. TAC expressed as mg of cyanidin-3-glucoside equivalents per 100 mL.

The anthocyanin content followed a similar pattern of degradation during storage (Figure 6A). The TAC decreased by ~62% in FWF-R1 (6.08 to 2.28 mg/100 mL) and ~64% in FWF-R2 (6.54 to 2.34 mg/100 mL) over 21 days (*p* ≤ 0.05). After digestion, the TAC levels were significantly reduced (*p* ≤ 0.05), with ~65–70% losses in both formulations (Figure 6B,C), indicating substantial anthocyanin degradation or transformation during gastrointestinal simulation.

### 3.9. Consumer Perception and Acceptability of Functional Fermented Beverages

**Figure 7:**
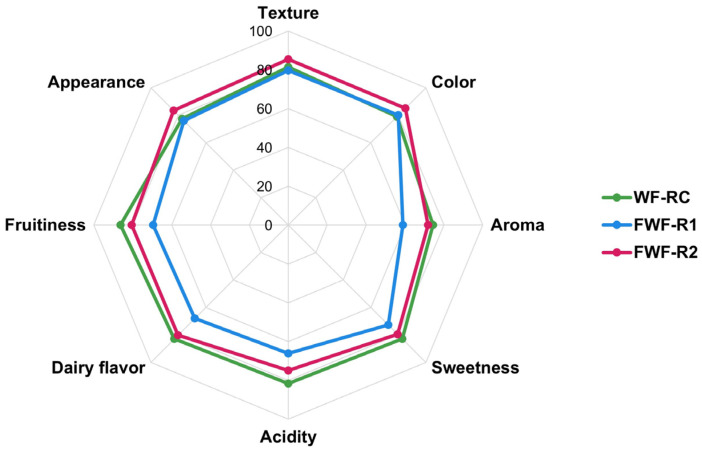
Sensory evaluation of fermented whey formulations with raspberry (FWF-R) and unfermented whey formulation containing raspberry as control (WF-RC). Data are expressed as acceptability index (%). FWF-R1 was fermented using mixed culture 1 (Mix 1), and FWF-R2 with mixed culture 3 (Mix 3). Microbial composition of each mixture is detailed in Table 1.

The sensory acceptability indices for the fermented whey beverages (FWF-R1 and FWF-R2) and the unfermented control (WF-RC) are shown in Figure 7. For their aroma, sweetness, acidity, dairy flavor, and fruitiness, WF-RC and FWF-R2 received comparable scores (*p* > 0.05), both significantly higher than those for FWF-R1. FWF-R2 also scored significantly better on texture (*p* ≤ 0.05). In terms of color, only FWF-R2 differed significantly from the control. For appearance, FWF-R2 was rated higher than both WF-RC and FWF-R1.

Overall, FWF-R2 was the preferred formulation, selected by 58% of participants vs. 42% for FWF-R1. Although only 14% of the panelists reported regular consumption of products targeting mental health, 74% considered mental health highly important, and 83% expressed willingness to purchase a product with such a claim.

## 4. Discussion

In this study, we developed fermented whey-based beverages with psychobiotic potential, using microbial strains selected for GABA production and favorable sensory attributes, and enriched with raspberry powder as a source of phenolics and fiber. A comprehensive approach was applied, including a matrix characterization, strain selection, formulation, assessment of physicochemical and microbial stability, evaluation of functional compound stability during storage and simulated digestion, and sensory analysis to determine their consumer acceptability as functional foods targeting mental health.

### 4.1. Whey as a Fermentation Substrate

The whey used in this study, obtained from the production of various cheeses, exhibited physicochemical properties typical of skim whey, with a high carbohydrate content and low levels of protein and fat (Table 2), consistent with previous reports [16,29]. Although the specific carbohydrate composition was not determined in this study, lactose is generally reported as the predominant carbohydrate in whey [16]. As documented in the literature, whey composition varies depending on the cheese type, animal species, diet, season, and processing conditions [16,18]. Its low WHC and viscosity reflects a reduced total solids content, as previously described [30]. These features confirm the suitability of whey as a fermentation substrate in terms of nutrient availability and physical characteristics.

Whey was selected not only for its technological and nutritional potential, but also for its relevance in sustainable food systems. As a major dairy industry by-product, whey poses serious environmental concerns due to its high organic load when improperly disposed [16,31]. Its biotransformation into functional fermented beverages offers a viable valorization strategy aligned with circular economy principles and food waste reduction targets [32].

### 4.2. Selection and Characterization of Microbial Strains for GABA-Enriched Fermented Whey Formulations

The BIOTEC strains used in this study were previously isolated from Mexican kefir and characterized for their probiotic potential, alongside the commercial strains LA3, GG, and 299v [11]. Here, each strain was individually assessed for its fermentative performance in non-supplemented pasteurized whey (Table 3). Most of the LAB exhibited significant growth (>8.5 log CFU/mL) and acidification capacity, consistent with prior studies using related strains. For instance, *L. lactis* strains reached 8.74–9.19 log CFU/mL and pH values of 3.72–4.02 in goat whey after 24 h [33]. Similarly, *L. mesenteroides* and *L. kefiri* have been used to produce exopolysaccharides in whey-based media [34,35]. Commercial strains like *L. acidophilus* and *L. rhamnosus* have also showed effective growth and acidification in dairy substrates, including whey [36,37]. *K. lactis*, known for lactose metabolism [38], showed limited growth in our whey system, with only a 1-log increase and minimal acidification. Nevertheless, despite its low biomass, *K. lactis* contributed to lactic acid formation, evident from the disappearance of whey’s sweetness (Table A1), in line with previous findings [39].

A central objective was to evaluate the psychobiotic potential of the strains based on their GABA production. GABA is synthesized via glutamic acid decarboxylation, catalyzed by glutamate decarboxylase (GAD) with pyridoxal-5′-phosphate as a cofactor [9]. GABA production can be classified as low (<0.5 mM), medium (0.5–2.1 mM), or high (>2.1 mM) [23]. According to this classification, most of the BIOTEC strains produced low-to-medium GABA levels, consistent with the limited glutamate naturally present in whey proteins [40]. In prior work, monosodium glutamate supplementation did not consistently enhance the GABA output, as only a few strains responded positively [11]. Thus, the whey remained non-supplemented in this study to assess its intrinsic production capacity. Given that GABA synthesis depends on multiple factors, including pH, temperature, substrate composition, and cofactor availability [41,42,43], this approach allowed for a meaningful evaluation of strain performance under realistic fermentation conditions. It should be noted that, although the GABase enzymatic assay used in this study is widely applied and standardized [23], it may be susceptible to interference from the matrix components in complex systems such as fermented beverages, potentially leading to an overestimation. More specific analytical techniques based on liquid chromatography could be employed in future studies to confirm the GABA quantification.

The sensory attributes guided strain selection (Table A1). *L. lactis* BIOTEC007, with balanced acidification and a yogurt-like aroma, was selected as the core starter. This strain has been shown to enhance the flavor and texture of dairy fermentations [44] and to engage in metabolic cross-feeding, particularly with glycine, alanine, and glutamic acid [45]. Based on this, we hypothesized that co-culturing with complementary strains could improve both the GABA production and sensory profiles.

From the acceptability testing (Table A2), three mixes were selected for GABA analysis. Mix 1 (*L. lactis* BIOTEC007 + LA3) and Mix 3 (*L. lactis* BIOTEC007 + *K. lactis* BIOTEC009 + *L. mesenteroides* BIOTEC012 + *L. kefiri* BIOTEC014 + LA3) achieved the highest GABA levels (Figure 2). Relative to the BIOTEC007 mono-culture, Mix 1 and Mix 3 increased GABA production by 2.0- and 2.2-fold, respectively. These results likely reflect synergistic metabolic interactions. In mixed cultures, several mechanisms may contribute to enhanced GABA production. The acidic environment created by LAB is essential for activating the GAD system. Additionally, cofactor modulation, such as an increased availability of pyridoxal-5′-phosphate, may further enhance enzyme activity. Cross-feeding interactions can also occur, where strains with higher proteolytic activity degrade proteins and release glutamate, serving as a substrate for GABA synthesis. Co-cultivation may also support cell viability and prolong the metabolically active phase, while certain strains may alleviate environmental stress (e.g., oxygen consumption by yeasts), creating more favorable conditions for LAB metabolism [41].

Similar findings have been reported: In a mulberry beverage, co-culturing *Lactiplantibacillus plantarum* BC114 and *Saccharomyces cerevisiae* SC125 increased GABA to 2.42 g/L, compared to 1.45 g/L and 1.03 g/L in mono-cultures [20]. Likewise, co-culturing *L. helveticus* and *L. rhamnosus* enhanced GABA in yogurt via GAD activity [46]. Recent transcriptomic analyses have provided mechanistic insights into this synergy. In a co-culture, *S. cerevisiae* SC125 upregulates the genes involved in GABA biosynthesis (*gad*, *gadA*) and glutamate transport (*glnH*, *glnM*) in *E. faecium* AB157, while downregulating the genes related to ATP synthesis and the arginine deiminase (ADI) pathway. This shift may increase intracellular glutamate availability and promote proton consumption via the GAD system, thereby enhancing GABA synthesis. Additionally, *S. cerevisiae* SC125 has been shown to induce *gad* expression in *E. faecium* through quorum sensing-regulated histidine kinase signaling [47]. These findings suggest that microbial communication and metabolic reprogramming may contribute to enhanced GABA production; however, further studies are needed to confirm whether these mechanisms are involved in the specific co-cultures used in this study.

To our knowledge, this is the first study comparing GABA production in mono- and co-cultures of kefir-derived strains in non-supplemented whey, underscoring their potential for use in developing functional beverages targeting mental health.

### 4.3. Functional Beverage Development, Nutritional Composition, and Physicochemical Stability During Storage

Following the selection of the most-promising co-cultures (Mix 1 and Mix 3) based on their GABA production and sensory profiles, a functional fermented whey beverage was developed incorporating health-promoting ingredients. The base formulation consisted of 75% fermented whey and 25% ultra-pasteurized skim milk, selected to enhance the sensory appeal and achieve a drinkable yogurt-like texture. This approach is consistent with previous strategies for improving the consumer acceptability of whey-based beverages [48]. Inulin (2.5%) was added for its widely recognized prebiotic and texturizing properties in dairy formulations [49,50,51]. Inulin promotes beneficial gut microbiota and SCFA production, supporting the gut–brain axis modulation [52,53]. Raspberry powder was included for its dietary fiber and bioactive compound content (Table 4), consistent with prior reports [54,55]. Its 2.5% addition provided meaningful bioactive levels without compromising the sensory acceptability. Similar doses (2–5%) have been used successfully in yogurt and whey-based products with fruit or vegetable powders [56,57].

The nutritional analysis (Table 5) showed no significant differences between FWF-R1 and FWF-R2, indicating that the addition of extra strains in FWF-R2 did not affect its composition post-fermentation. Comparable results have been reported in other whey–milk beverages, where strain differences did not alter the composition initially, although minor changes may occur over time due to lactose fermentation and protein hydrolysis [19]. The fat (0.13%) and protein (1.24%) contents of our beverages are intermediate compared to collagen-enriched whey drinks (0.02% fat, 0.94% protein) [58] and probiotic whey beverages (1.86% fat, 3.75% protein) [19]. The fiber content (0.97–1.12%) reflects the contributions from the inulin and raspberry powder, similar to findings for beverages enriched with acacia fiber [59] or microalgae [60].

The physicochemical parameters remained largely stable over 21 days of refrigerated storage, with only minor variations in the viscosity and WHC (Table 6). This aligns with previous studies reporting minimal changes in acidity and the stability of fermented whey products during storage [19,61,62]. The gradual increase in viscosity is characteristic of fiber- or polysaccharide-rich formulations, where the interactions between milk proteins and added components like inulin or raspberry fiber enhance the viscosity and WHC [63]. Our WHC values (28–36%) are comparable to those observed in fruit-enriched whey drinks (9–21%) [63]. Minor differences between the formulations may stem from strain-specific exopolysaccharide production, which influences the textural attributes [51].

### 4.4. Microbial Viability and Bioactive Compound Stability in Functional Beverages During Storage and After Digestion

Both FWF-R1 and FWF-R2 maintained stable microbial viability over 21 days of refrigerated storage at 4 °C, with final counts exceeding 8.5 log CFU/mL (Figure 3). These results align with previous studies reporting the sustained viability of *L. rhamnosus* GG in guava whey beverages for up to 35 days [64], and of *L. acidophilus* and *L. casei* in oat bran–raspberry drinks over 21 days [57]. Similarly, stable counts (≥7 log CFU/mL) of various LAB in whey-based beverages with juice concentrates were found [65]. Following the simulated gastrointestinal digestion, both formulations retained high viability (>8 log CFU/mL). FWF-R1 showed no significant viability loss post-digestion, while FWF-R2 experienced only minor reductions (0.3–0.5 log CFU/mL), supporting their potential as probiotic carriers with intestinal functionality [66]. These values compare favorably with previous reports, which observed greater losses for *K. lactis* in whey-based drinks [67] and 0.5–1.0 log reductions in LAB viability during digestion [68]. The protective effect may be attributed to the food matrix and the presence of dietary fibers, which enhance survival during digestion [69]. Nonetheless, the intrinsic acid and bile tolerance of each strain, linked to their proton regulation capacity, also plays a key role [70,71]. The strains used here had previously shown intestinal resilience in MRS medium [11].

A key finding is the continued increase in GABA concentration during storage (Figure 4), suggesting sustained microbial GABA synthesis post-fermentation. This behavior mirrors the results for refrigerated GABA-enriched yogurts [72]. The GABA levels increased by 93% in FWF-R1 and by 208% in FWF-R2 over the 21-day period, both reaching values classified as high production [23]. This contrasts with other studies reporting GABA degradation in whey-based drinks [73], likely due to microbial GABA metabolism via the Krebs cycle. Our findings suggest that the microbial combinations and the inclusion of functional ingredients created favorable conditions for GABA retention. The simulated digestion resulted in GABA recovery rates of approximately 40–45%, which are within the range reported for fermented foods and reflect the matrix-dependent nature of GABA bioaccessibility [74,75]. Since GABA is a non-proteinogenic amino acid, it is not degraded by pepsin or pancreatin, and its losses are likely due to interactions with the matrix or gut microbiota [72,76]. Some studieshave reported complete GABA retention in kombucha-type beverages [77].

Depending on the storage period, the GABA concentrations prior to digestion ranged from 14.2 to 27.5 mg for FWF-R1 and from 15.6 to 48.0 mg for FWF-R2 per 100 mL serving. These values fall within the range reported for fermented foods developed through optimized fermentation processes, including yogurt, fermented milk, and mulberry beer (4–100 mg/100 mL) [78]. Several clinical and preclinical studies support the functional relevance of GABA concentrations comparable to or even lower than those found in our beverages, although the effective doses may vary depending on the food matrix and the specific health outcome evaluated [79,80]. For instance, a preclinical study using bean sprout fermented milk containing 24.1 mg GABA/100 mL showed increased levels of hippocampal neurotransmitters associated with antidepressant effects [81]. In terms of anxiety, a human trial demonstrated that the intake of GABA-enriched chocolate (28 mg GABA/10 g) significantly reduced psychological stress [82]. Similarly, GABA-fortified oolong tea containing only 2.01 mg GABA/200 mL led to a measurable reduction in acute stress levels in healthy individuals [83]. Although these studies evaluated orally ingested GABA, our post-digestion concentrations (equivalent to 5.0–10.6 mg/100 mL for FWF-R1 and 8.0–21.0 mg/100 mL for FWF-R2, depending on the storage period) indicate that a relevant proportion of the GABA remained bioaccessible, supporting the potential functionality of the developed beverages. Given the limited data on GABA stability and bioaccessibility in whey-based systems, further in vivo studies are needed to confirm GABA absorption and the physiological outcomes following consumption.

Regarding the bioactive compounds, both beverages showed a ~22–23% decline in the TPC during storage (Figure 5), like the reports on berry-enriched dairy products [24]. This contrasts with previous findings, which reported no significant phenolic losses in similar matrices [57]. The decline may be attributed to oxidation or microbial activity. Remarkably, the simulated digestion increased the TPC by 4.7–5.1-fold, likely due to the acid- and enzyme-mediated release of bound phenolics [84], as also reported in aronia kefir [85] and raspberry purée [86]. The anthocyanins decreased by 62–64% during storage (Figure 6), reflecting their well-known sensitivity to light, oxygen, pH, and temperature [87,88]. Post-digestion, the TAC further declined by 65–70%, consistent with its degradation under gastrointestinal conditions [84,86]. This reduction was likely due to their hydrolysis and conversion into smaller phenolic compounds. Despite these losses, the initial TPC and TAC levels remained within functionally relevant ranges [89].

Although the TPC increased after digestion, the marked degradation of TAC may have reduced its specific contribution to the antioxidant capacity, as anthocyanins are recognized for their potent radical-scavenging activity [90]. Additionally, anthocyanin-rich foods have been linked to mental health benefits. For instance, mulberry milk containing 3.43 mg/100 mL TAC reduced anxiety and depressive symptoms after a daily intake of one or two 180 mL servings [91]. This concentration aligns with the initial TAC levels in our beverages (6.54–2.28 mg/100 mL, depending on the formulation and storage time). However, the degradation observed during digestion may attenuate these potential benefits. Importantly, the quantitative reduction in the TAC does not necessarily imply a loss of bioactivity, as certain degradation products, such as protocatechuic acid, may retain or even enhance its functional properties [92]. Further studies are warranted to validate the in vivo functionality of and explore strategies to improve anthocyanin stability in fermented matrices.

### 4.5. Sensory Acceptability and Psychobiotic Implications

The sensory evaluation indicated that FWF-R2 was the preferred fermented formulation, selected by 58% of participants. This preference may be attributed to its higher sensory ratings across most attributes, particularly sweetness, fruitiness, and texture (Figure 7). Positive perceptions of sweetness and fruitiness are well-known drivers of consumer acceptance of dairy–fruit products [93,94], while undesirable changes in texture or acidity can negatively affect appeal [95]. Moreover, FWF-R2 received significantly higher scores for its overall appearance and color, which likely enhanced its visual attractiveness.

Although only 14% of the respondents reported currently consuming products marketed for mental health, 83% expressed a willingness to purchase them if supported by clear functional claims. This suggests a promising market opportunity for psychobiotic-positioned beverages. However, effective communication strategies are needed to convey these benefits, as public awareness of the link between fermentation and psychological health remains limited [96].

From a psychobiotic standpoint, FWF-R2 appears particularly promising. It combines LAB and yeast strains with probiotic potential, exhibits greater GABA accumulation, and retains functionally relevant levels of phenolics and anthocyanins, bioactives known to influence the gut–brain axis. These results support future studies to evaluate its psychobiotic potential in animal or human models, especially given the rising interest in dietary strategies for mental health support [97].

## 5. Conclusions

This study provides scientific evidence supporting the development of fermented whey-based beverages enriched with 2.5% raspberry and 2.5% inulin as functional foods aimed at promoting mental well-being. Through a targeted microbial selection strategy focused on GABA production and favorable sensory attributes, we formulated beverages with high probiotic viability, sustained GABA accumulation, stable levels of bioactive compounds, and good consumer acceptance. The FWF-R2 formulation, comprising *L. lactis* BIOTEC007, *K. lactis* BIOTEC009, *L. mesenteroides* BIOTEC012, *L. kefiri* BIOTEC014, and *L. acidophilus* LA3, exhibited superior performance, with the highest GABA content and overall consumer preference.

These findings not only demonstrate the technological and functional feasibility of the proposed formulations but also highlight their potential as psychobiotic dietary interventions. By leveraging agro-industrial by-products, this approach can contribute to both environmental sustainability and public health. Furthermore, the integration of multiple functional components (probiotics, prebiotic fibers, and polyphenol-rich fruit) offers a promising model for the development of next-generation functional beverages targeting the gut–brain axis.

## Figures and Tables

**Table 1:** Microbial composition and inoculum proportions (% *v*/*v*) of the microbial consortia used for whey fermentation ^1^.

Microorganism	Mix 1	Mix 2	Mix 3
*Lactococcus lactis* BIOTEC007	2%	2%	2%
*Kluyveromyces lactis* BIOTEC009	—	—	0.5%
*Leuconostoc pseudomesenteroides* BIOTEC012	—	1%	0.5%
*Lentilactobacillus kefiri* BIOTEC014	—	—	0.5%
*Lactobacillus acidophilus* LA3	2%	1%	0.5%

^1^ Each consortium was inoculated at a total concentration of 4% (*v*/*v*).

**Table 2:** Physicochemical properties and proximate composition of pasteurized skim whey.

Parameter	Value ^1^
Physicochemical properties	
pH	5.70 ± 0.02
Titratable acidity (g/L)	1.68 ± 0.07
Viscosity (mPa·s)	42.75 ± 1.07
Water-holding capacity (%)	1.33 ± 0.10
Proximate composition (g/100 g fresh whey)	
Protein	0.67 ± 0.03
Fat	0.07 ± 0.03
Carbohydrates	4.59 ± 0.04
Dietary fiber	0.00 ± 0.00
Ash	0.51 ± 0.00

^1^ Means ± standard deviation.

**Table 3:** Growth and pH changes during whey fermentation by microorganisms isolated from Mexican kefir and commercial probiotics ^1^.

Microorganism	Initial Count (Log CFU/mL)	Final Count (Log CFU/mL)	pH After Fermentation
Pasteurized whey (non-inoculated control)	<1	<1	5.70 ± 0.02 ^a^
*Lactococcus lactis* BIOTEC006	6.68 ± 0.16	6.91 ± 0.12	5.48 ± 0.06 ^b^
*Lactococcus lactis* BIOTEC007	6.91 ± 0.40	8.88 ± 0.27 *	4.62 ± 0.01 ^e^
*Lactococcus lactis* BIOTEC008	6.87 ± 0.06	8.85 ± 0.51 *	5.00 ± 0.01 ^c^
*Kluyveromyces lactis* BIOTEC009	6.92 ± 0.28	7.72 ± 0.28 *	5.02 ± 0.01 ^c^
*Kluyveromyces lactis* BIOTEC010	6.69 ± 0.38	6.35 ± 0.17	5.71 ± 0.00 ^a^
*Leuconostoc pseudomesenteroides* BIOTEC011	6.32 ± 0.12	6.10 ± 0.58	5.73 ± 0.03 ^a^
*Leuconostoc pseudomesenteroides* BIOTEC012	7.61 ± 0.03	8.55 ± 0.16 *	4.60 ± 0.01 ^e^
*Lentilactobacillus kefiri* BIOTEC013	6.84 ± 0.49	8.51 ± 0.10 *	4.64 ± 0.04 ^e^
*Lentilactobacillus kefiri* BIOTEC014	7.11 ± 0.16	7.96 ± 0.10 *	4.99 ± 0.01 ^c,d^
*Lentilactobacillus parakefiri* BIOTEC015	5.64 ± 0.40	7.98 ± 0.09 *	4.94 ± 0.01 ^d^
*Lactobacillus acidophilus* LA3	7.82 ± 0.11	8.89 ± 0.19 *	4.44 ± 0.02 ^f^
*Lacticaseibacillus rhamnosus* GG	7.79 ± 0.35	8.86 ± 0.06 *	4.03 ± 0.01 ^g^
*Lactiplantibacillus plantarum* 299v	7.88 ± 0.14	8.86 ± 0.19 *	4.04 ± 0.04 ^g^

^1^ Means ± standard deviation. The strains with a significant increase in concentration after fermentation (*p* ≤ 0.05) are marked with an asterisk. Different lower-case letters indicate significant differences (*p* ≤ 0.05) in the final pH among the microorganisms. Non-inoculated pasteurized whey was included as a control to assess the baseline pH. CFU: colony-forming units.

**Table 4:** Physicochemical properties, proximate composition, and bioactive content of raspberry fruit and powder.

Parameter	Value ^1^
Physicochemical properties	
Apical caliber (cm)	2.38 ± 0.35
Equatorial caliber (cm)	2.19 ± 0.18
Weight (g)	5.39 ± 1.60
Moisture content (g/100 g fresh weight)	84.27 ± 0.53
Soluble solids (°Brix at 25 °C)	10.10 ± 0.10
pH	2.81 ± 0.01
Titratable acidity (g citric acid/100 g fresh weight)	0.51 ± 0.09
Proximate composition (g/100 g dry weight)	
Protein	7.67 ± 0.21
Fat	2.53 ± 0.05
Carbohydrates	65.51 ± 0.31
Dietary fiber	20.82 ± 0.18
Insoluble dietary fiber	16.80 ± 0.21
Soluble dietary fiber	4.02 ± 0.10
Ash	3.47 ± 0.41
Total bioactive compounds (mg/g dry weight)	
Total phenolic content ^2^	9.21 ± 0.61
Total monomeric anthocyanin content ^3^	2.64 ± 0.14

^1^ Means ± standard deviation. ^2^ Expressed as mg of gallic acid equivalents/g dry weight. ^3^ Expressed as mg of cyanidin-3-glucoside equivalents/g dry weight.

**Table 5:** Proximate composition of fermented whey formulations with raspberry (FWF-R).

Parameter (g/100 g Fresh Weight)	FWF-R1 ^1^	FWF-R2 ^1^
Protein	1.24 ± 0.08	1.24 ± 0.01
Fat	0.13 ± 0.00	0.13 ± 0.02
Carbohydrates	8.98 ± 0.05	9.12 ± 0.06
Dietary fiber	1.12 ± 0.09	0.97 ± 0.08
Insoluble dietary fiber	0.68 ± 0.06	0.56 ± 0.06
Soluble dietary fiber	0.44 ± 0.03	0.41 ± 0.02
Ash	0.57 ± 0.04	0.66 ± 0.09

^1^ Means ± standard deviation. There were no significant differences (*p* ≤ 0.05) between FWF-R1 and FWF-R2 for any parameter. FWF-R1 was fermented using mixed culture 1 (Mix 1), and FWF-R2 with mixed culture 3 (Mix 3). Microbial composition of each mixture is detailed in Table 1.

**Table 6:** Physicochemical properties of fermented whey formulations with raspberry (FWF-R) during refrigerated storage ^1^.

Property	Formulation	Day 0	Day 1	Day 7	Day 14	Day 21
pH	FWF-R1	3.71 ± 0.01 ^b,B^	3.74 ± 0.01 ^b,A^	3.75 ± 0.01 ^b,A^	3.74 ± 0.02 ^b,A^	3.74 ± 0.01 ^b,A^
	FWF-R2	3.78 ± 0.01 ^a,B^	3.82 ± 0.01 ^a,A,B^	3.86 ± 0.02 ^a,A^	3.85 ± 0.03 ^a,A^	3.84 ± 0.03 ^a,A^
Titratable acidity (% lactic acid)	FWF-R1	1.25 ± 0.02 ^a,A^	1.27 ± 0.06 ^a,A^	1.25 ± 0.01 ^a,A^	1.24 ± 0.02 ^a,A^	1.26 ± 0.01 ^a,A^
	FWF-R2	1.19 ± 0.02 ^b,A^	1.21 ± 0.02 ^a,A^	1.19 ± 0.02 ^b,A^	1.19 ± 0.09 ^a,A^	1.20 ± 0.07 ^a,A^
Soluble solids (°Brix at 25 °C)	FWF-R1	9.90 ± 0.10 ^b,B^	10.33 ± 0.06 ^b,A^	10.34 ± 0.06 ^b,A^	10.47 ± 0.15 ^b,A^	10.37 ± 0.25 ^b,A^
	FWF-R2	10.50 ± 0.03 ^a,B^	10.60 ± 0.10 ^a,B^	10.63 ± 0.06 ^a,B^	10.90 ± 0.02 ^a,A^	10.83 ± 0.05 ^a,A^
Viscosity (mPa·s)	FWF-R1	266.93 ± 11.30 ^a,C^	347.10 ± 37.43 ^a,B^	384.43 ± 2.40 ^a,B^	405.17 ± 1.14 ^a,A^	408.50 ± 3.27 ^a,A^
	FWF-R2	226.30 ± 3.42 ^b,D^	301.23 ± 6.80 ^b,C^	301.57 ± 5.03 ^b,C^	321.00 ± 3.25 ^b,B^	344.27 ± 3.52 ^b,A^
Water-holding capacity (%)	FWF-R1	28.75 ± 0.71 ^a,B^	33.49 ± 2.96 ^a,A^	36.59 ± 2.53 ^a,A^	34.48 ± 2.84 ^a,A^	34.37 ± 0.88 ^a,A^
	FWF-R2	29.82 ± 0.26 ^a,B^	33.71 ± 0.43 ^a,A^	35.27 ± 1.46 ^a,A^	34.50 ± 0.73 ^a,A^	33.73 ± 0.56 ^a,A^

^1^ Means ± standard deviation. Different lowercase letters within same day indicate significant differences (*p* ≤ 0.05) between formulations. Different uppercase letters within same formulation indicate significant differences (*p* ≤ 0.05) between storage days. FWF-R1 was fermented using mixed culture 1 (Mix 1), and FWF-R2 with mixed culture 3 (Mix 3). Microbial composition of each mixture is detailed in Table 1.

## Data Availability

The original contributions presented in the study are included in the article, further inquiries can be directed to the corresponding author.

## Abbreviations

The following abbreviations are used in this manuscript:

C3G	Cyanidin-3-glucoside equivalents
CFU	Colony-forming units
CNS	Central nervous system
FWF-R	Fermented whey formulation with raspberry
GABA	Gamma-aminobutyric acid
GAE	Gallic acid equivalents
GMBA	Gut–microbiota–brain axis
LAB	Lactic acid bacteria
LM17	M17 broth supplemented with 0.5% (*w*/*v*) lactose
MRS	Man–Rogosa–Sharpe broth
SCFA	Short-chain fatty acids
TAC	Total anthocyanin content
TPC	Total phenolic content
WHC	Water-holding capacity
YPD	Yeast extract–dextrose–peptone medium

## Appendix A

An internal sensory screening was conducted by a panel of nine food product specialists (five females and four males) from the Food and Biotech Group at Tecnologico de Monterrey, Campus Guadalajara. Each panelist evaluated the mono-cultures fermented in whey by tasting 20 mL of each pure-culture fermented whey sample. The evaluations included qualitative assessments of the aroma and flavor, summarized in Table A1. Subsequently, both the mono-cultures and mixed cultures were evaluated quantitatively using an acceptability index for acidity, sweetness, texture, and overall perception, as defined in Section 2.8 of the main manuscript. This activity was conducted as part of the general sensory analysis approved by protocol CA-EIC-2406-02.

**Table A1:** Qualitative sensory attributes of fermented whey using individual strains.

Microorganism	Qualitative Sensory Attributes
*Lactococcus lactis* BIOTEC006	Mild yogurt-like aroma with a pleasant, unsweet clean taste.
*Lactococcus lactis* BIOTEC007	Light, fruity notes in aroma and a yogurt-like, slightly acidic taste.
*Lactococcus lactis* BIOTEC008	Pronounced yogurt aroma and noticeable acidic flavor.
*Kluyveromyces lactis* BIOTEC009	Evident gas formation; alcoholic aroma and dry, non-sweet flavor.
*Kluyveromyces lactis* BIOTEC010	Gas release and a mild alcoholic flavor profile.
*Leuconostoc pseudomesenteroides* BIOTEC011	Sweet taste with a characteristic whey-derived flavor.
*Leuconostoc pseudomesenteroides* BIOTEC012	Slightly fruity aroma with a flavor reminiscent of soft cheese.
*Lentilactobacillus kefiri* BIOTEC013	Sharp, highly acidic, and noticeably astringent taste.
*Lentilactobacillus kefiri* BIOTEC014	Mild dairy aroma with a clean, neutral taste.
*Lentilactobacillus parakefiri* BIOTEC015	Neutral-to-pleasant flavor with low sweetness perception.
*Lactobacillus acidophilus* LA3	Fruity aroma with intense acidic flavor; slightly astringent.
*Lacticaseibacillus rhamnosus* GG	Yogurt-like aroma; pronounced acidic taste and lingering aftertaste.
*Lactiplantibacillus plantarum* 299v	Faint acidic-like smell; intense acidic flavor.

**Table A2:** Acceptability indices (%) for fermented whey formulations prepared with mono and mixed microbial cultures.

**Culture (Mono or Mixed) ^1^**	**Acidity**	**Sweetness**	**Texture**	**Overall Perception**
BIOTEC007	25	100	63	85
BIOTEC009	56	75	100	52
BIOTEC012	56	70	75	48
BIOTEC014	28	60	63	52
LA3	81	60	100	81
BIOTEC007 + BIOTEC009	88	55	88	48
BIOTEC007 + BIOTEC012	78	50	88	78
BIOTEC007 + BIOTEC014	25	55	69	67
BIOTEC007 + LA3	100	65	81	89
BIOTEC007 + BIOTEC009 + BIOTEC014	66	55	94	67
BIOTEC007 + BIOTEC007 + BIOTEC012 + BIOTEC014	44	75	69	81
BIOTEC007 + BIOTEC012 + LA3	84	55	94	100
BIOTEC007 + BIOTEC009 + BIOTEC012 + BIOTEC014 + LA3	75	55	94	96

^1^ Full microorganism names are provided in Table A1.
